# Gamma radiation crosslinking of PVA/myrrh resin thin film for improving the post-harvest time of lemon fruits†

**DOI:** 10.1039/d1ra09360f

**Published:** 2022-02-16

**Authors:** Tarek M. Mohamed, Mohamed S. Attia, Gharieb S. El-Sayyad, Rasha M. Fathy, Ahmed I. El-Batal

**Affiliations:** Polymer Chemistry Department, National Center for Radiation Research and Technology (NCRRT), Egyptian Atomic Energy Authority (EAEA) P.O Box 29 Cairo Egypt; Botany and Microbiology Department, Faculty of Science (Boys), Al-Azhar University Cairo Egypt; Drug Microbiology Lab., Drug Radiation Research Department, National Center for Radiation Research and Technology (NCRRT), Egyptian Atomic Energy Authority (EAEA) P.O Box 29 Cairo Egypt Gharieb.S.Elsayyad@eaea.org.eg

## Abstract

Preparation of a thin film of polyvinyl alcohol (PVA)/myrrh natural resin using a low gamma irradiation dose (1 kGy) was investigated towards increasing the post-harvest time of lemon fruit. Different analytical techniques, such as Fourier transform infrared (FTIR) spectroscopy, scanning electron microscopy (SEM), energy-dispersive X-ray (EDX) spectroscopy, and mapping techniques were used to characterize the prepared thin film. This investigation was carried out to evaluate the effect of different concentrations of myrrh as an edible coating in prolonging shelf life and preserving the quality of lemon fruits (*Citrus aurantifolia*). Lemons were immersed directly in PVA solution containing 1%, 2%, and 3% concentrations of myrrh and then stored at ambient (25 ± 1 °C) and low (4 ± 1 °C) temperatures. The disease severity, acidity, total soluble solids (TSS), and ascorbic acid contents were tested after the coating with the PVA/myrrh thin film at different temperatures (4 °C and 25 °C) for different storage times (7 and 14 days). The application of different concentrations of the synthesized PVA/myrrh thin film (1%, 2%, and 3%) significantly reduced green mold disease symptoms and disease severity in the lemon fruits. The acidity value (pH value) was the lowest for the 2% myrrh treatment after 7 °C days at 25 °C, followed by the 1% myrrh treatment under the same conditions. The highest TSS was observed after the treatment for 7 days at 25 °C, with a value of 8.1 g dL^−1^. A high ascorbic acid concentration (33.5 mg dL^−1^) was noted after coating the lemons with the 1% PVA/myrrh thin film for 7 days at 25 °C. The results show that the application of a PVA/myrrh thin film extends the shelf-life and maintains the quality of lemon fruits by decreasing the levels of evaporation from the fruits and loss of weight due to the delay of the complete ripening stage of the lemon fruits.

## Abstract 2

Preparation of a thin film of polyvinyl alcohol (PVA)/myrrh natural resin using a low gamma irradiation dose (1 kGy) was investigated. The results show that PVA/myrrh thin film extends the shelf-life and maintains the quality of lemon fruits.

## Graphical abstract

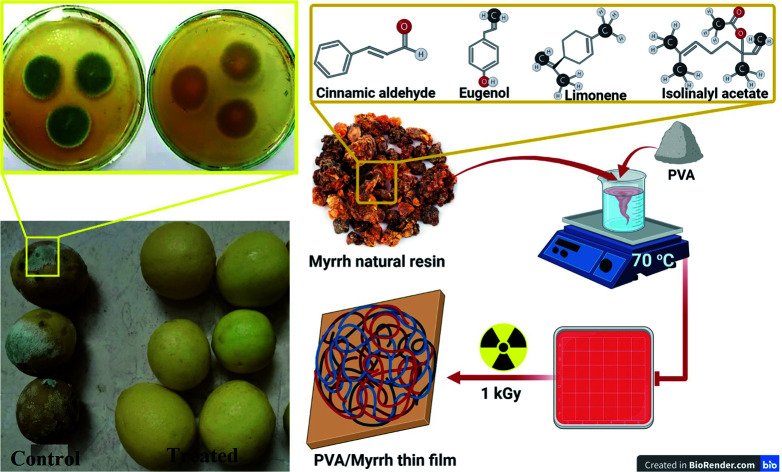

## 1 Introduction

Citrus is a type of subtropical and tropical fruit, which includes oranges, lemons, limes, grapefruit, tangerines, and pomelos.^1^ The origin of the lemon is unknown, though lemons are thought to have first grown in Asia, in Assam (a region in northeast India), northern Burma or China.^2^ A study of the genetic origin of the lemon reported it to be a hybrid between a bitter orange (sour orange) and citron.^3^ Lemons belong to the family Rutaceae,^4^ and are widely cultivated in tropical and subtropical regions.^5^ Lemons are known for their beautiful appearance, pleasing flavor, and excellent food qualities.^6^ Therefore, there has been increased interest in the food safety and nutrition of lemons amongst consumers. This increase in health consciousness has led to a demand for organic fruits and vegetables and healthy preservation techniques.

Significant causes of postharvest losses of citrus fruits are lack of maintenance of orchards, faulty harvesting methods, mishandling of produce, mold growth and rotting, fading and weight loss, loss of firmness, improper means of distribution, and improper storage facilities.^6,7^

Damage to the fruit mainly occurs because they are biologically active and carry out transpiration, respiration, ripening and other biochemical activities.^8–10^ According to some reports, 25% to 80% of harvested fresh fruits and vegetables may be lost due to spoilage worldwide.^11,12^ Hence, research is being carried out to increase the shelf-life of fruits and vegetables without loss in their nutritional content by identifying and studying the reason for the quality deterioration of these commodities and providing a feasible solution to this ubiquitous problem.^8,13,14^

Green mold triggered by *Penicillium digitatum* is the principal postharvest disease that affects citrus production worldwide through transfer and the marketplace.^15,16^ Attempts to control postharvest diseases have been ongoing for a long time. However, a solution has not yet been reached, and the control of citrus fruits conditions is still dependent on fungicides, such as imazalil or thiabendazole.^17^ However, using fungicides on a large scale to control postharvest diseases in the long term reduces their effectiveness due to fungicide-resistant pathogens.^18,19^ The effect of pre-harvest sprays of chitosan on postharvest decay and quality of strawberries stored at 3 °C and 13 °C was investigated by Reddy *et al.*^20^ The data on decay and ripening characteristics provided quantitative evidence that chitosan compensates for higher storage temperature and protects against lower-quality fruit from the second harvest. Atghia *et al.*^21^ investigated the potential impact of salicylic acid as a natural defence inducer on radical growth, spore germination, and disease development afflicted by *P. digitatum*. *In vivo* assays confirmed that salicylic acid remarkably reduced the lesion diameter on the lemon fruits before inoculation with the tested pathogens. A new paper published by Yongmei *et al.*^22^ details the potential of the endophyte *Bacillus subtilis* L1-21, isolated from healthy citrus plants, as a biocontrol bacteria possesses activity against the pathogen *P. digitatum*. The results suggested that the production of antifungal compounds and the colonization potential of *B. subtilis* L1-21 are required against the postharvest *P. digitatum* pathogen on citrus fruit.

Myrrh is one of the oldest known medicines, widely used by ancient Egyptians.^23^ Myrrh is derived from the Arabic and Hebrew word mur, which means bitter.^24^ The meeting is the trade name for Arabian myrrh.^24^ The various reports on myrrh's therapeutic value and uses attracted our attention use it to produce a thin protective film.^25,26^ Myrrh is an oleo-gum-resin obtained from the stem of different species of Commiphora.^27^ It is a reddish-brown mass, covered with a brownish-yellow dust.^24^ It has a bitter, acrid taste and a balsamic odour,^28^ and it forms an emulsion with water.^24^ Myrrh is made up of 2–8% volatile oil (myrrhol), 23–40% resin (myrrhin), 40–60% gum, and 10–25% bitter compounds.^29,30^

This article, we believe, reports for the first time the preparation of a thin film of myrrh that has the advantages of being safe, biocompatible, low cost and easy preparation and handling. As mentioned above, myrrh consists of water-soluble gum, alcohol-soluble resins and volatile oil. The gum contains polysaccharides and proteins, while the volatile oil is composed of steroids, sterols and terpenes.^31^ When these components of myrrh solution were mixed with polyvinyl alcohol (PVA) and subjected to a low gamma irradiation dose (1 kGy), a crosslinked network (oligomer) was formed, which was applied to lemon fruits *via* their immersion in the prepared oligomer solution and then dried at room temperature to form a thin film membrane that covers the lemon fruits and protected them from environmental conditions. The film presents a safe, eco-friendly, cost-effective method of preserving lemon fruits at different temperatures (4 °C, and 25 °C) for different storage times (7, and 14 days), with its complete screening and encouraging data showing its feasibility for possible real-life application.

## 2 Materials and methods

### 2.1. Materials

PVA was purchased from Qualikems Ltd, India and myrrh was obtained from Rgab Alattar market, Al-Houssen zone, Cairo, Egypt. Co-60 gamma rays at a dose rate of 1.68 kGy h^−1^ were used at the NCRRT, Cairo, Egypt.

### 2.2. Preparation of the materials

PVA (2%) was dissolved in hot distilled water at 70 °C with continuous stirring until completely dissolved. After that, different concentrations of myrrh extract of 1, 2, and 3% were added to the PVA solution (w/w%) with stirring, and finally, this mixture subjected to 1 kGy gamma rays to produce the oligomer material that could be readily applied to lemon fruits.

The radiolysis of water by ionizing radiation produces particles such as photons, electrons or ions, giving rise to new chemical species. The radiolysis of water leads to both oxidizing (H_2_O_2_, O_2_, OH˙, O_2_˙, O_2_˙^−^) and reducing (H_2_, H˙, e_aqu_^−^) species, among which some species are non-stable (solvated electrons), while others are stable molecules (H_2_O_2_, O_2_, H_2_).^32,33^

**Fig. 1:**
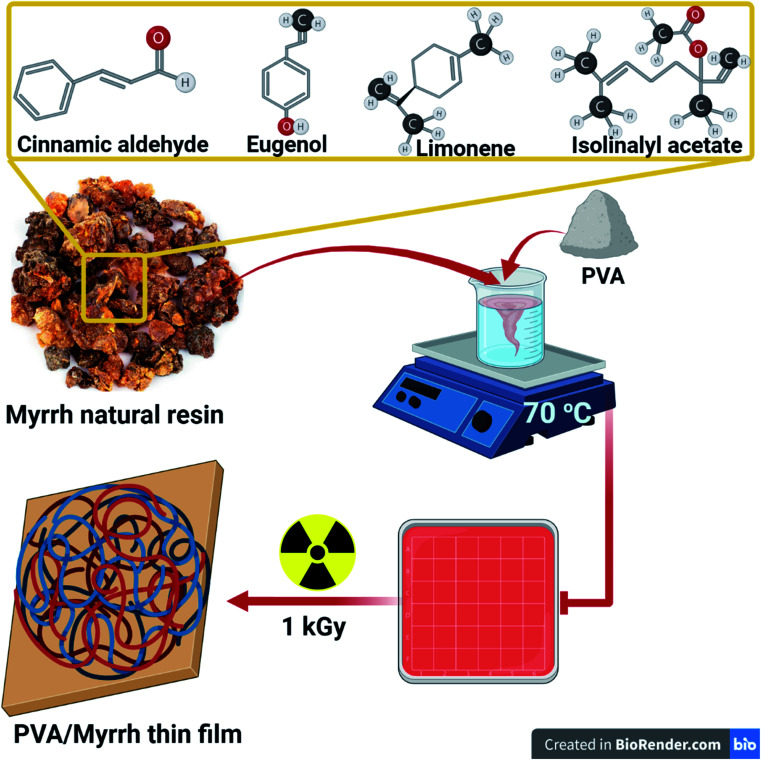
Schematic representation of the components and preparation of the PVA/myrrh thin film by gamma irradiation.

Also, gamma radiation leads to the cleavage of π bonds of unsaturated compounds in the myrrh resin, which leads to the formation of other free radicals linked to other radicals produced from to the radiolysis of water and the construction of the polymer, as shown in Fig. 1.

### 2.3. Characterization methods

#### 2.3.1. Fourier-transform infrared (FTIR) spectroscopy

The FTIR spectra of myrrh natural resin, PVP, and PVA/myrrh thin film were recorded using attenuated total reflectance-Fourier transform infrared (ATR-FTIR) spectroscopy on a Vertex 70 FTIR spectrometer equipped with a HYPERION™ series microscope (Bruker Optik GmbH, Ettlingen, Germany) over the range of 4000–400 cm^−1^ at a resolution of 4 cm^−1^. The software OPUS 6.0 (Bruker) was used for data processing, which was baseline-corrected using the rubber band method with the exclusion of CO_2_ bands.

#### 2.3.2. Surface morphology determination and elemental mapping imaging estimation

Surface morphology examination of the as-synthesized myrrh natural resin, PVP, and PVA/myrrh thin film was conducted using a Zeiss SEM Ultra 60 field-emission scanning electron microscope (FESEM) operated at an acceleration voltage of 5 kV. Elemental mapping was used to estimate the elemental distribution of the synthesized myrrh natural resin, PVA, and PVA/myrrh thin film and was investigated by coupled SEM/EDX (BRUKER, Nano GmbH, D-12489, 410-M, Germany) mapping analysis.

### 2.4. Fruit treatment and preparation of samples for coating

The lemon fruits (*Citrus aurantifolia*) used in this study were harvested from trees in a commercial garden located in Kalybia, Egypt, and sorted based on size uniformity, color, absence of damage, fungal contamination and lack of physical injuries. The collected lemon samples were placed on separate sterilized foam plates, surface disinfected by dipping for 2 min in a 10% sodium hypochlorite solution, and rinsed twice with distilled water. Before use, they were isolated at room temperature, and their dry weights measured.^34^ After drying for 1 h, the fruits were randomized into treatment groups, then wounded at four equidistant points at an equatorial site. Each wound was 5 mm in diameter and 4 mm in depth. 24 h before inoculation, the fruits were dipped into PVA/myrrh solution for 10 seconds and air-dried for 30 min under a fan to dry them. Fruits dipped in distilled water following the same procedures were used as controls. In this study, PVA was used as a substrate and carrier for the myrrh components, as the properties of PVA make it a suitable membrane in dry form, which is dependent only on the active ingredients and materials of the myrrh resin.

### 2.5. Isolation and maintenance of pathogens

Highly aggressive isolates of *Penicillium digitatum* were isolated from infected lemon fruits according to a method presented by Katan *et al.*,^35^ and identified macroscopically according to their morphology and culture features.^36^ The fungal cells were grown on a specific medium such as potato dextrose agar (PDA) for 6 days and incubated at 24 ± 1 °C. The incubated cultures were transported again onto PDA for 6 days at 24 ± 1 °C to induce fungal sporulation. Finally, fungal conidial solutions were developed as outlined by El Guilli *et al.*^37^ The density of the fungal spores was determined using a hemocytometer and set to 10^7^ spore mL^−1^. Additionally, the fungal pathogen was established using the pathogenicity experiment described by Hibar *et al.*^38^ Koch's postulate was used to confirm the pathogen as the causal agent of *Penicillium digitatum* (green mould) on the lemon fruits.^39^

### 2.6. Preparation of fruits and antifungal activity assay

Lemon fruits (*Citrus aurantifolia*) were harvested from a commercial garden (Kalybia, Egypt) and immediately moved to the laboratory. The fruits were selected free of wound sand rots and were, as much as possible, homogeneous in terms of physiological maturity stage and size. Fruits were washed with distilled water and surface-disinfected by spraying with ethanol (70%). In detail, we brought the fruits to the laboratory, disinfected and sterilized them, and after that, artificial infection was achieved according to the method described in a published paper,^40^ where 2 mm in depth holes were made on the fruits at three points using a sterile nail. Fungal pathogen suspension (1 mL) was injected into the hole, and then the fruits were left for 4 h to establish fungus spores in the holes. We divided fruits into four groups (0, 1%, 2%, and 3% myrrh) to dip polluted fruits for 10 min in myrrh solutions. After applying the treatments, the fruits were kept in a plastic bag and stored at 10 °C for three weeks. Data recording was performed every week, and the polluted fruits were removed from the bags.

### 2.7. Storage conditions

The fruits were divided into two groups with 6 foam dishes containing nine fruits. The first group was stored at room temperature (≈25 °C), and the second stored at 4 °C. The fruits in the dishes were treated according to the following: control (without any treatment) at 4 °C or 25 °C, coated with myrrh I (1%), coating with synthetic myrrh II (2%), and coated with synthetic myrrh III (3%).

### 2.8. Data and parameters

#### 2.8.1. Disease severity

Disease severity represents the percentage diseased portion of the infected fruit and was measured based on estimation by eye. Disease severity was scored as per the method described by Sivakumar *et al.*,^41^ using the scale 1 = 0% of fruit surface rotten; 2 = 1–25%; 3 = 26–50%; 4 = 51–75%, and 5 = 76–100%.

#### 2.8.2. Acidity

The pH of the homogenized treated and untreated lemons was measured using a pH meter.^42^

#### 2.8.3. Total soluble solids

The total soluble solids (TSS) content of the lemon fruits was determined using a refractometer (Atago) and performed as described by You *et al.*^43^ The refractometer was calibrated with distilled water to give a 0 Brix reading for each measurement and the results expressed as g dL^−1^. A few drops of lemon sap were then placed in the compartments of the refractometer before reading.

#### 2.8.4. Vitamin C (ascorbic acid) content

Precisely 3.0 mL of citrus juice was added to a 10 mL graduated cylinder using an eyedropper, with the volume and type and brand of citrus juice noted. One or two drops of indophenol solution were then added to the graduated cylinder using the eyedropper. The graduated cylinder was then shaken to mix the citrus juice and indol-phenol mixture.^44^ Indophenol blue was added until the reaction mixture changed color to a blue or purple color. The volume of the reaction mixture was recorded at the endpoint.^45^ The reaction mixture was discarded into a waste beaker, then the graduated cylinder was rinsed with about 1 mL of citrus juice.^44^ The titration was then repeated, and if the total volume of the reaction mixture for two titrations was in agreement to within ±0.2 mL, then a different juice was tested.^45^ If the total volume of the reaction mixture for the two titration was not within ±0.2 mL, then a third titration was carried out.^44,45^

#### 2.8.5. Statistical analysis

All the aforementioned conclusions were carried out in triplicate, and average values and standard deviations were noted. Analysis of variance (ANOVA) was used to measure mean separations and the least significant difference (LSD) method at *α* = 0.05 was conducted using statistical software.^46^

## 3 Results and discussion

### 3.1. Characterization of the myrrh coating

#### 3.1.1. FTIR spectroscopy analysis

**Fig. 2:**
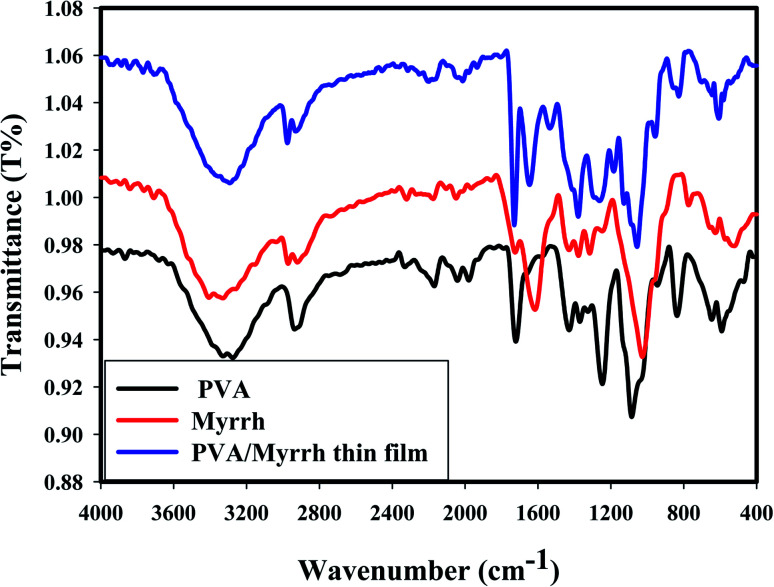
FTIR analysis of PVA, myrrh natural resin, and the PVA/myrrh thin film.

FTIR spectroscopy is a tool that is used to determine organic components, including chemical bonds, to identify and investigate the structure of materials.^32^Fig. 2 shows the FTIR spectra of PVA, myrrh resin and PVA/myrrh resin film, respectively.

As shown from the FTIR spectra, the broad absorption bands at around 3274 cm^−1^, 3330 cm^−1^, and 3292 cm^−1^ correspond to the O–H stretching vibrations of PVA, myrrh resin and PVA/myrrh, respectively. The sharp bands at 1326 cm^−1^, 1316 cm^−1^, and 1379 cm^−1^ can be attributed to the O–H bending vibrations of PVA, myrrh and PVA/myrrh, respectively. The absorption band at 2936 cm^−1^ in the spectrum of PVA are due to the C–H stretching, and bands for the same group are present in the spectrum of 2920 cm^−1^ for myrrh and at 2930 cm^−1^ for PVA/myrrh.^47^ The bands of the carbonyl group (C=O) can be observed at 1725 cm^−1^ for myrrh and 1730 cm^−1^ for PVA/myrrh.^48^ The C–O stretching vibrations are observed for PVA at 1085 cm^−1^, 1026 cm^−1^ for myrrh, and 1056 cm^−1^ for PVA/myrrh.^49,50^ The sharp absorption bands at 1616 cm^−1^ and 1646 cm^−1^ can be attributed to the stretching vibration of C=C in some components in the myrrh resin and PVA/myrrh, respectively.^49,50^ Some new characteristic bands shown in the spectrum of the PVA/myrrh film can be attributed to the formation of hydrogen bonds between PVA and some components of the myrrh resin.^51^

Comparing the spectra of PVA only and PVA/myrrh, some bands are slightly shifted and reduced in intensity due to the interactions and coupling between the PVA and myrrh components. The peak at 680 cm^−1^, which can be assigned to metal–oxygen (M–O) stretching vibrations,^52^ corresponds to the deformation vibrations of M–O.^53^

#### 3.1.2. SEM and EDX analysis

**Fig. 3:**
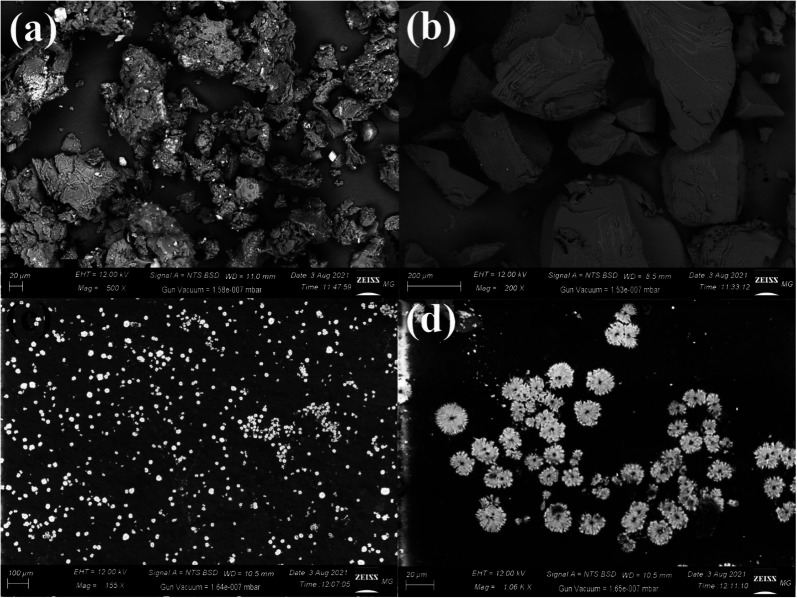
SEM imaging of (a) pure myrrh, (b) pure PVA, (c) the PVA/myrrh thin film, and (d) magnified image of the synthesized PVA/myrrh thin film.

SEM images of pure PVA, pure myrrh, and the synthesized PVA/myrrh thin film are illustrated in Fig. 3, where each layer was imaged and analyzed separately. Typical SEM was used to determine the appearance of the prepared PVA/myrrh thin film and its external morphology. At the same time, EDX analysis is a technique that was used in the elemental examination or chemical validation of the synthesized PVA/myrrh thin film sample.^54–57^ The image of pure myrrh presented in Fig. 3a shows the mixed elements of the components of myrrh, and Fig. 3b shows the SEM imaging analysis of pure PVA, showing its solid block appearance without any observed impurities. The synthesized PVA/myrrh thin film presented in Fig. 3c shows its good sheet-like appearance with loaded myrrh as bright particles equally distributed on the surface of the PVA thin film, as shown in the magnified part presented in Fig. 3d.

**Fig. 4:**
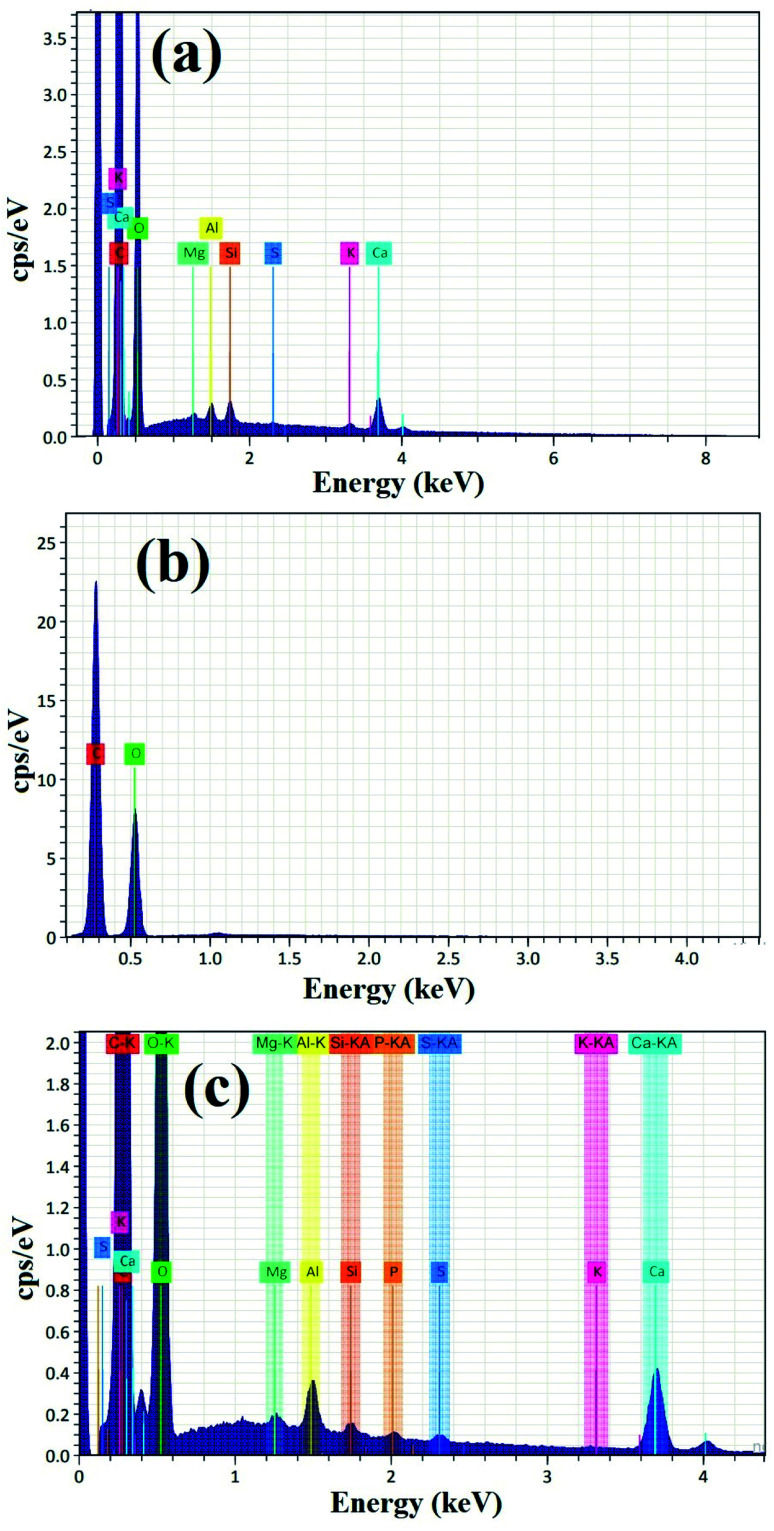
EDX elemental analysis of (a) pure myrrh, (b) pure PVA, and (c) the PVA/myrrh thin film.

Fig. 4 shows the EDX analysis of pure PVA, pure myrrh, and the synthesized PVA/myrrh thin film. The EDX analysis of pure myrrh (Fig. 4a) confirms that it possesses all of the elements that make up the structure of myrrh, with high purity and showing the presence of C, O, Mg, Al, Si, S, K, and Ca atoms. Additionally, Fig. 4b shows the EDX analysis of pure PVA, which confirms that it exhibits uniform distribution and high purity, confirming the presence of C and O atoms. Finally, the synthesized PVA/myrrh thin film (Fig. 4c) can be seen to have a uniform distribution. EDX elemental analysis was used to confirm the presence of two layers of PVA with the coated myrrh which confirms the same atoms presented in the tested sample with high purity. The corresponding EDX elemental analysis confirms the presence of all of the particles that make up the thin film, with high purity.

#### 3.1.3. Chemical mapping of the synthesized PVA/myrrh thin film elements

**Fig. 5:**
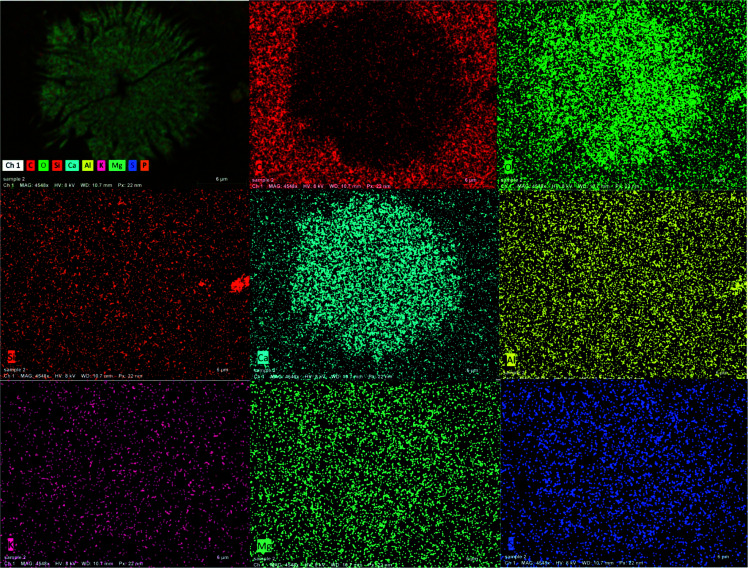
Elemental composition/mapping of the synthesized PVA/myrrh thin film.

The elemental mapping images of the synthesized PVA/myrrh thin film are displayed in Fig. 5. The images can be identified as C, O, Si, Ca, Al, K, Mg, S, and P for the synthesized PVA/myrrh thin film. Fig. 5 confirms the synthesis of the PVA/myrrh thin film in terms of the appearance of C, O, Si, Ca, Al, K, Mg, S, and P in its EDX analysis. C atoms are also present due to the imaging holder and as a component of PVA. The elemental mapping images confirmed the formation of highly distributed myrrh elements, as seen by their brightness across the PVA thin film. Finally, it is worth mentioning that the bright color of condensed ‘Ca’ atoms indicated that the Ca layer was the main elemental constituent of the myrrh active ingredient.

### 3.2. Fungal culture macroscopic examination

When first grown, *Penicillium digitatum* colonies are yellow-green but ultimately turn olive due to conidial production^58^ (Fig. S1-a†). The reverse of the plate can be pale or barely tinted brown^58^ (Fig. S1-b†). *P. digitatum* presents filamentous vegetative growth, generating narrow, septate hyphae.^59^ The hyphal cells are haploid, and the conidia are borne on a stalk called a conidiophore that emerges from aerial hyphae, a soil-embedded network of hyphae.^60^ The conidiophore is asymmetrical, with smooth and thin walls. The conidiophore can radiate into three rami during growth to create bi-verticillate or tri-verticillate.^61^ At the end of each ramus, additional branches called metulae are found.^62^ At the distal ending of each metula, conidium-bearing networks called phialides form.^63^ The conidia delivered, in turn, are fluffy with a shape that can vary from round to cylindrical and are created in chains, with the youngest at the base of each chain.^59^

### 3.3. Effect of coating materials

#### 3.3.1. Disease severity

**Fig. 6:**
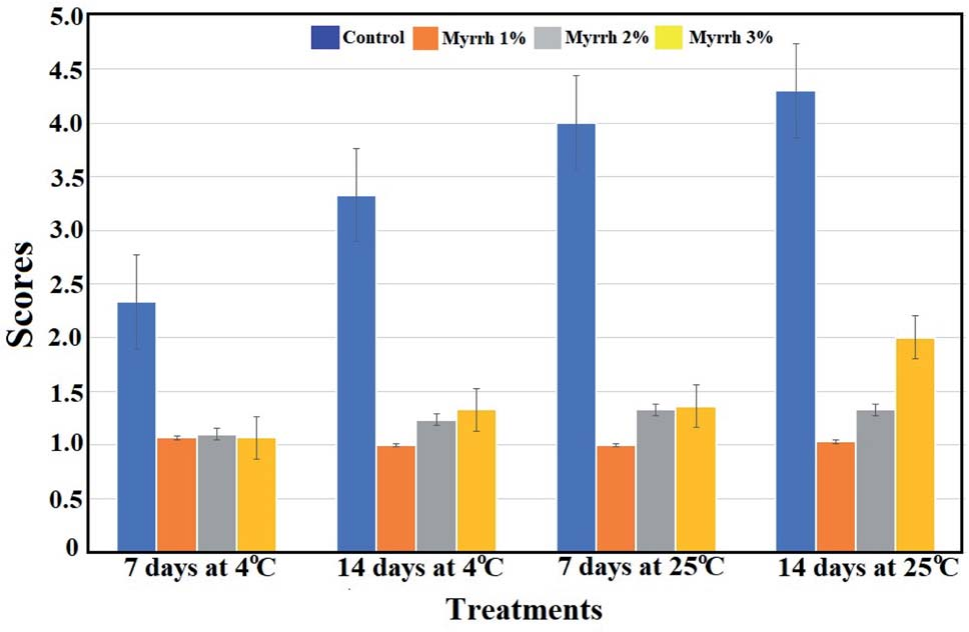
Effect of the synthesized PVA/myrrh thin film on disease severity (DS); DS was calculated using the scale 1 = 0% of fruit surface rotten; 2 = 1–25%; 3 = 26–50%; 4 = 51–75%; and 5 = 76–100%.

It is evident from Fig. 6 that the application of all of the concentrations of synthesized PVA/myrrh thin film (1%, 2%, and 3%) significantly reduced the green mold disease symptoms and disease severity caused by *P. digitatum* compared to the untreated control lemon fruits.

**Fig. 7:**
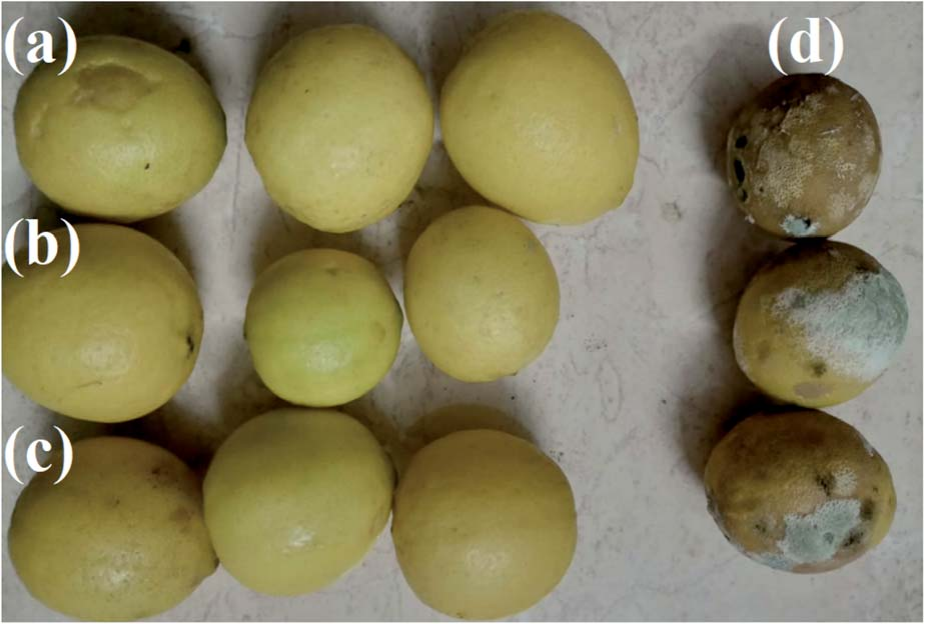
Effect of the synthesized PVA/myrrh thin film on lemon fruits disease symptoms, where (a) is coated with 1% PVA/myrrh thin film, (b) is coated with 2% PVA/myrrh thin film, (c) is coated with 3% PVA/myrrh thin film, and (d) the untreated control.

Myrrh concentrations 1 (1%) and 2 (2%) were the best treatments and reduced disease severity during the different storage conditions. The first guide to controlling the occurrence of resistance towards the pathogen of fruits treated with the synthesized PVA/myrrh thin film was the reduced disease severity and high protection against the pathogen infection, as shown in Fig. 7.

#### 3.3.2. Fruit chemical composition

**Fig. 8:**
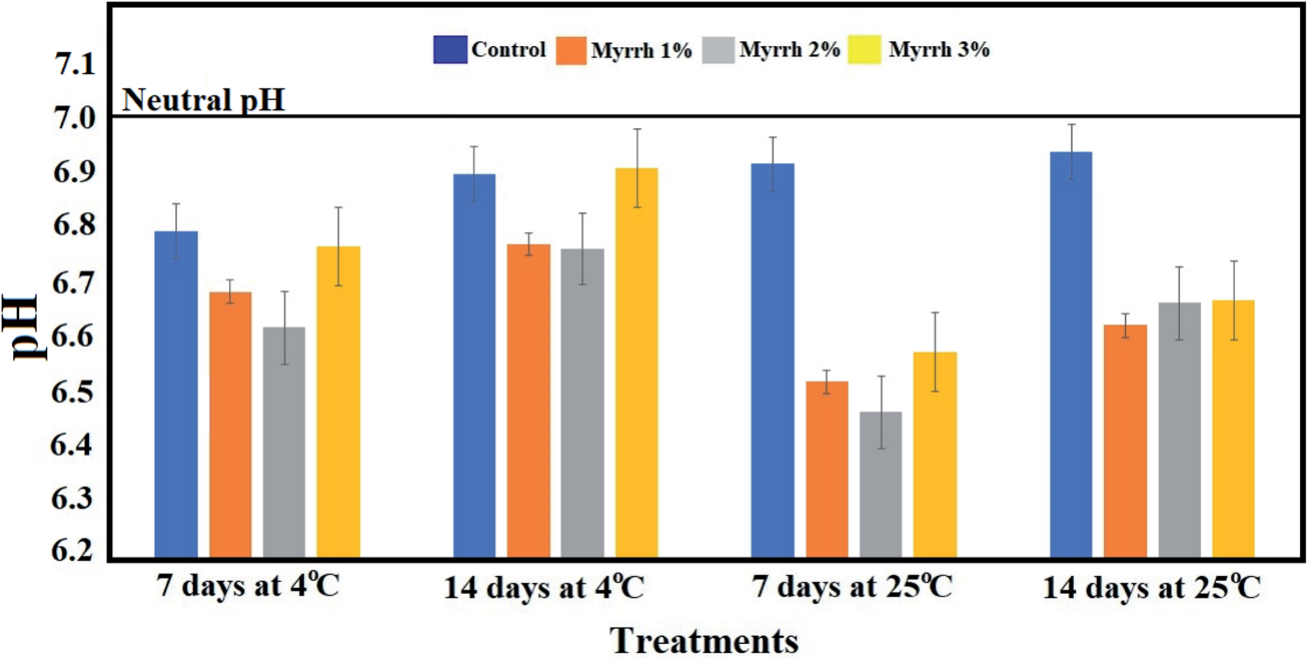
Effect of the synthesized PVA/myrrh thin film on titratable acidity during the storage of lemon fruits.

Potency evaluation of the protective functions of the synthesized PVA/myrrh thin film on lemon chemical composition was investigated. As shown in Fig. 8, the acidity value (pH value) was low for the 2% myrrh treatment after 7 °C days at 25 °C (room temperature), followed by 1% myrrh under the same conditions, which confirms that the use of the synthesized PVA/myrrh thin film improves the post-harvest time of lemon fruits at room temperature as a cost-effective approach. The acidity values of the lemon also showed a significant decrease throughout the storage time, regardless of the coating of the PVA/myrrh thin film. Fig. 8 shows that the pH of the fruits treated with the synthesized PVA/myrrh thin film was positively-affected despite the long storage period (14 and 7 days) and the temperature (4 °C or 25 °C). The lowest pH of the fruits was noted in the treatment with concentrations 1% and 2% of the synthesized PVA/myrrh thin film at temperatures of 4 °C and 25 °C after 7 and 14 days, while the pH of fruits treated with 3% myrrh was not affected. These results show that the substance, with its content of antioxidants and its anti-fungal efficacy, preserved the external structures of the lemon fruits, which led to an improvement or maintenance of the pH throughout the storage period and the change in temperature, which is one of the essential features that defines the quality of lemon fruits. These results are consistent with other reports in the literature,^64–66^ which found that the acidity decreased after all treatments with the increase in the length of the storage period and change in temperature.

**Fig. 9:**
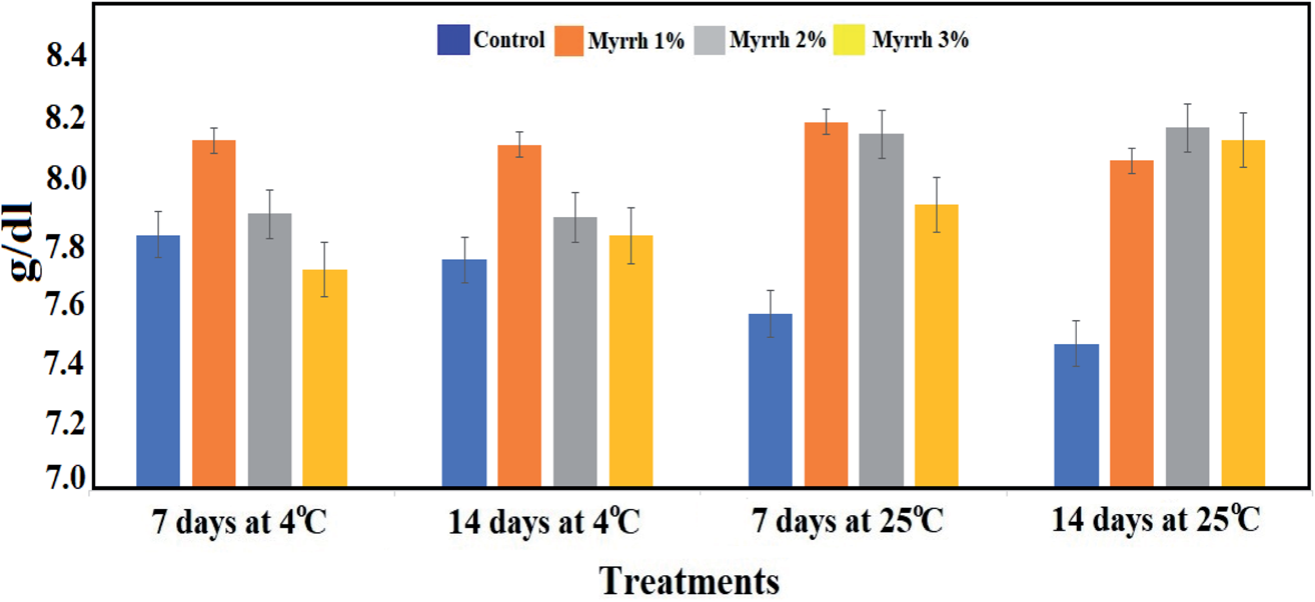
Effect of the synthesized PVA/myrrh thin film on the total soluble solids (TSS) content during the storage of lemon fruits.

Focusing on the total soluble solids content of lemon fruits during the storage period and the different temperatures (4 °C or 25 °C), Fig. 9 shows a significant decrease in the total soluble solids content in the untreated fruits throughout the storage period. At the same time, the highest content of total soluble solids was in the fruits treated with the 1% and 2% synthesized PVA/myrrh thin-films at 4 °C or 25 °C over storage periods of 7 and 14 days compared to the untreated control. These results are due to the extent of the effect of the synthesized PVA/myrrh thin film on the characteristics of the outer shell from environmental factors and the loss of its chemical contents. The results of this analysis are in concurrence with those in recently published studies.^37,40,66^

**Fig. 10:**
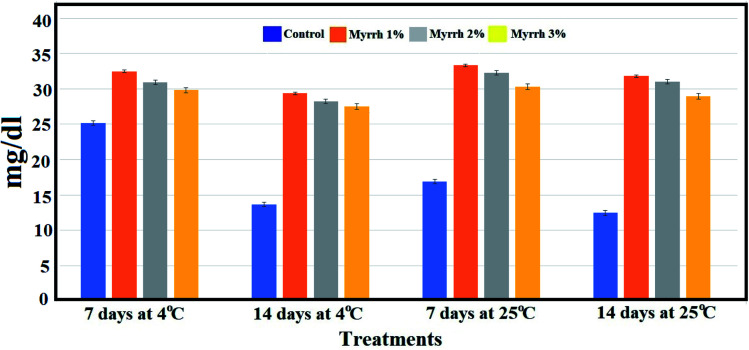
Effect of the synthesized PVA/myrrh thin film on the ascorbic acid content during the storage of lemon fruits.

It was found that both storage time and coating time had a significant effect on the ascorbic acid content of the fruits.^67^ The ascorbic acid content increased during the storage time and tended to be higher for the PVA/myrrh thin-film coated fruit than uncoated fruit (Fig. 10). The 1% and 2% PVA/myrrh thin films were the best treatments in preserving and increasing ascorbic acid content, which improves the quality of the lemon fruits in long-term storage periods at different temperatures. These results are consistent with those of Zagzog and Mohsen,^68^ who found that ascorbic acid starts to break down immediately after the picking of the fruit and breaks down continuously upon an extension in the storage period. It was shown that the reason for the low percentage of ascorbic acid in untreated lemon fruits is the rapid transformation of ascorbic acid into dehydrogenated ascorbic acid in the presence of the ascorbinase enzyme.^69,70^

The present study was aimed at protecting lemon fruits from a fungal infection that reduces the quality of the fruits. The results indicated different responses to the concentrations used to improve the quality of lemon fruits in terms of lowering their pH and increasing vitamin C content (ascorbic acid), and this, of course, has no negative impact on the taste of the fruits as the lemon fruits contain many organic acids that are responsible for their pungent taste, such as malic acid and citric acid.^71,72^

## 4 Conclusion

This study was concerned with the preparation of a safe, biocompatible, low-cost, and easy prepared and handled PVA/myrrh thin film. Gamma radiation leads to the cleavage of the p bonds of the unsaturated compounds in the myrrh resin, which leads to the formation of another free radical linked with other radicals produced from the radiolysis of water and leads to the polymer's appearance. FTIR spectroscopy revealed the appearance of new characteristic bands in the spectrum of the PVA/myrrh thin film attributed to the formation of hydrogen bonds between PVA and some of the components of the myrrh resin. SEM images of the synthesized PVA/myrrh thin film show its good sheet-like appearance with loaded myrrh observed as bright particles equally distributed on the surface of the PVA thin film. It is evident from the disease severity tests that the application of all of the concentrations of the synthesized PVA/myrrh thin films (1%, 2%, and 3%) significantly reduced the green mold disease symptoms and disease severity caused by *P. digitatum* compared to the untreated control lemon fruits. The pH of the fruits treated with the synthesized PVA/myrrh thin film was positively affected despite their long storage period (14 and 7 days) or change in temperature (4 °C or 25 °C). The lowest pH of the fruits was noted in the treatments with concentrations of 1% and 2% of the synthesized PVA/myrrh thin film at temperatures of 4 °C and 25 °C for 7 and 14 days, while the pH of the fruits treated with a concentration of 3% myrrh were not affected. The ascorbic acid content of the fruits increased during their storage time and tended to be higher for the PVA/myrrh thin-film coated fruit than the uncoated fruit. Overall, the results show that the application of a PVA/myrrh thin film extends the shelf-life and maintains the quality of lemon fruits.

## Conflicts of interest

The authors declare that they have no conflict of interest.

## Supplementary Material

RA-012-D1RA09360F-s001
