# Supplementary material for: Gamma radiation crosslinking of PVA/myrrh resin thin film for improving the post-harvest time of lemon fruits
Source: RSC Adv. 2022 Feb 16;12(9):5619–28. doi: 10.1039/d1ra09360f (PMC8981498; doi:10.1039/d1ra09360f)
Supplement: RA-012-D1RA09360F-s001 [file RA-012-D1RA09360F-s001.pdf]

Electronic Supplementary Material (ESI) for RSC Advances.
This journal is © The Royal Society of Chemistry 2022

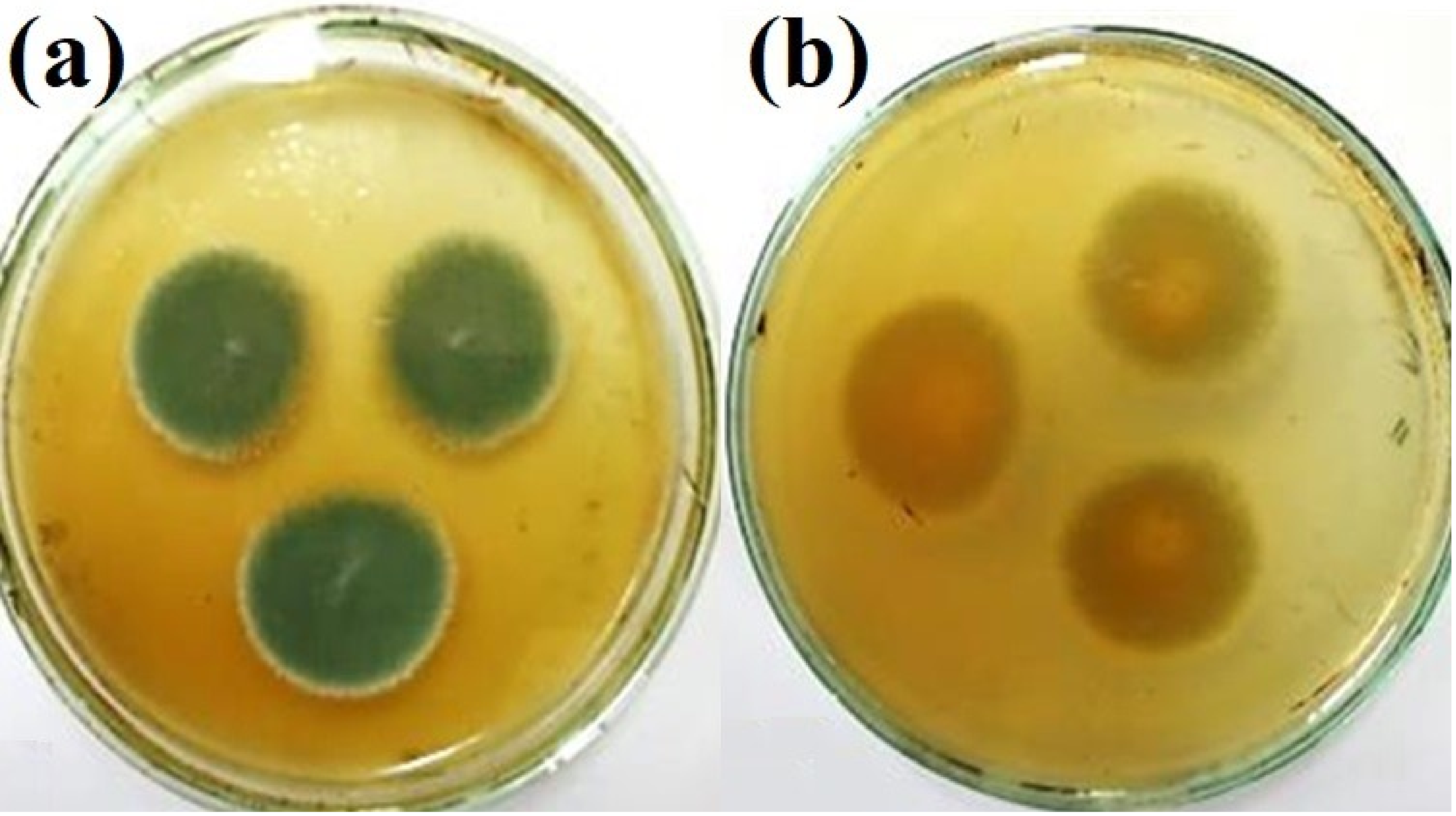

Figure S1: Colony of *Penicillium digitatum* on PDA grown for 6 days and incubated at 24 ±1°C showing the culture characteristics (a), and Reverse colony of *P. digitatum* (b).
